# Application and Challenge of Metalloporphyrin Sensitizers in Noninvasive Dynamic Tumor Therapy

**DOI:** 10.3390/molecules29204828

**Published:** 2024-10-11

**Authors:** Jiacheng Ouyang, Dan Li, Lizhen Zhu, Xiaoyuan Cai, Lanlan Liu, Hong Pan, Aiqing Ma

**Affiliations:** 1 Research Center of Nano Technology and Application Engineering, Dongguan Innovation Institute, Guangdong Medical University, Dongguan 523808, China; 2 Guangdong Key Laboratory of Nanomedicine, CAS-HK Joint Lab of Biomaterials, CAS Key Laboratory of Biomedical Imaging Science and System, Shenzhen Institutes of Advanced Technology (SIAT), Chinese Academy of Sciences, Shenzhen 518055, China; 3 The Second Affiliated Hospital, Guangdong Medical University, Zhanjiang 523808, China

**Keywords:** metalloporphyrin, sensitizers, photodynamic therapy, sonodynamic therapy, ROS generation

## Abstract

Dynamic tumor therapies (mainly including photodynamic therapy (PDT) and sonodynamic therapy (SDT)) offer new approaches to cancer treatment. They are often characterized by their noninvasive nature, high selectivity, and low toxicity. Sensitizers are crucial for dynamic therapy. Developing efficient sensitizers with good biocompatibility and controllability is an important aim in dynamic therapy. Porphyrins and metalloporphyrins attract great attention due to their excellent photophysical properties and low cytotoxicity under non-light. Compared to porphyrins, metalloporphyrins show greater potential for dynamic therapy due to their enhanced photochemical and photophysical properties after metal ions coordinate with porphyrin rings. This paper reviews some metalloporphyrin-based sensitizers used in photo/sonodynamic therapy and combined therapy. In addition, the probable challenges and bottlenecks in clinical translation are also discussed.

## 1. Introduction

Cancer is one of the leading causes of death worldwide, and its treatment continues to face significant challenges [1,2,3,4]. The popular treatment methods include surgery, chemotherapy, radiotherapy, and immunotherapy. Although each method shows certain therapeutic effects, serious side effects often accompany them [5,6,7,8]. For instance, chemotherapy aims to quickly kill cancer cells using chemicals, but it also inevitably damages normal cells, leading to obvious side effects. Immunotherapy has demonstrated significant clinical efficacy against malignant tumors in the blood system by activating the antitumor immune response; however, its therapeutic effect on solid tumors remains limited [9]. Additionally, the introduction of too many cytotoxic T cells into the body during immunotherapy can trigger a severe cytokine storm, resulting in fever, low blood pressure, organ toxicity, and other side effects [10]. Therefore, improving the therapeutic effect on tumors while minimizing side effects remains a significant challenge.

Noninvasive dynamic therapies, such as photodynamic therapy (PDT) and sonodynamic therapy (SDT), provide new opportunities for tumor treatment, especially for solid tumors [11,12,13,14]. After absorbing sensitizers, the tumor can be irradiated by light with specific wavelengths or ultrasound waves, which excite the sensitizers to generate reactive oxygen species (ROS, such as ·OH, ^1^O_2_, and ^−^O_2_) to kill the tumor cells [15,16]. Because the sensitizers are of low or zero toxicity in the absence of light and ultrasound irradiation, noninvasive dynamic therapies exhibit excellent antitumor potential. PDT has a big drawback in that light penetration is not strong enough to reach deep tumor lesions, limiting its effects [17]. Therefore, PDT always shows efficiency for superficial tumors. SDT is derived from PDT and aims to address the issue of poor light penetration [18,19]. Due to the deep penetration properties of ultrasound in soft tissue, SDT shows superior application prospects compared to PDT.

**Figure 1:**
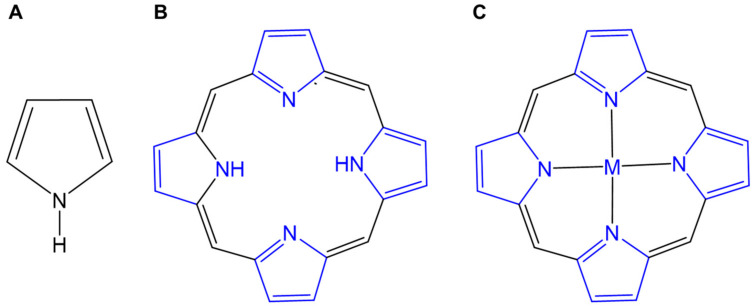
Typical structures, i.e., (**A**) pyrrole, (**B**) porphyrin, consisting of four pyrrole rings joined by methane bridges, and (**C**) metalloporphyrin (M = Mn, Fe, Cu, Zn, and so on).

Sensitizers are a key to tumor dynamic therapies. Porphyrins and their derivatives (such as hematoporphyrin, protoporphyrin, and ATX-70) were first used in PDT and later in sonodynamic therapy SDT [20,21]. Porphyrins are a unique class of organic molecules with heterocyclic tetrapyrrole structures, and are commonly found in nature. Structurally, porphyrin rings consist of four pyrrole units connected in a coplanar manner by four methylene bridges, forming a planar macrocyclic structure (Figure 1). The internal cavity of porphyrins can coordinate metal ions to form metalloporphyrins. Porphyrins and metalloporphyrins have a highly conjugated π-bond system, which contributes to their efficient absorption of visible light, making them promising sensitizers [22,23]. Moreover, compared to free porphyrins, the electrocatalytic and photophysical properties of metalloporphyrins can be significantly adjusted and even enhanced [24]. Studies have shown that metalloporphyrins have significant advantages, including high singlet oxygen yield, easy excitation, and a broad range of reactions [25]. To date, various metalloporphyrins have been utilized in biomedical applications and therapeutics [26,27,28,29]. In this review, we provide an overview of the basic principles of PDT and SDT, along with recent research on the metalloporphyrins as sensitizers in these therapies. Finally, the future prospects and challenges of using metalloporphyrins as sensitizers are also discussed, offering theoretical insights for their application in sensitizers and other medical fields.

## 2. The Possible Therapeutic Mechanism of PDT and SDT

Sufficient ROS are the basis of PDT and SDT. In general, ROS generation results from energy (triggered by light or ultrasound) transfer to sensitizers and electron transition [30,31]. These ROS increase oxidative stress levels in tumor cells, causing genetic and cell membrane damage, thus leading to apoptosis of cancer cells. However, due to the different energy sources of PDT and SDT, the mechanism of ROS generation is not exactly the same.

**Figure 2:**
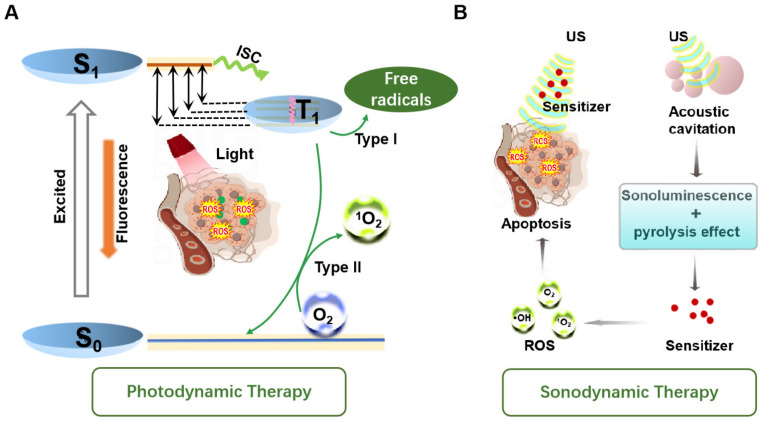
The possible mechanisms of PDT (**A**) and SDT (**B**).

In PDT, after the sensitizer absorbs photon energy, electron transition of sensitizer occurs from the ground state (S_0_) to a higher singlet energy state (S_1_) [32]. Following internal conversion, the excited electrons can return to the ground state through fluorescence emission. Alternatively, they can transition to a longer-lived triplet excited state (T_1_) via a non-radiative spin electron transition. During this process, excited state sensitizers oxide surround reducing substances to form free radicals (·OH) or superoxide ions (^−^O_2_) and other ROS species through intersystem crossing (ISC). This is often called a type I reaction [33]. When the photosensitizers return from the excited T_1_ state to the ground state, the released energy is transferred to surrounding O_2_, converting it to singlet oxygen, known as ^1^O_2_. This is also called a type II reaction. Usually, the type I and type II reactions take place at the same time (Figure 2A) [34].

Unlike the wave–particle duality of light, ultrasound is a mechanical wave. The unique effects caused by ultrasound, including cavitation and acoustic luminescence, cannot be ignored. In fact, there is no clear definition for the process of ROS production in SDT. One proposed mechanism is similar to PDT: the sonosensitizers transition to an excited state and induce ROS production as they return to the ground state. Conversely, the cavitation effect is widely accepted as the primary mechanism for ROS generation under ultrasound excitation [35]. When ultrasonic waves pass through a liquid environment or tissue, they cause microbubbles to oscillate in the sound field [36]. As the sound pressure increases, the microbubbles eventually implode, releasing large amounts of light (sonoluminescence) and heat [37]. Sonoluminescence can stimulate the photochemical reaction of sonosensitizers, causing them to enter an excited state (S_1_), where they react with dissolved oxygen molecules or other substrate molecules to produce free radicals and superoxide ions. Meanwhile, the energy released by sonosensitizers as they return from the high-energy state to the ground state is transferred to oxygen molecules, producing ^1^O_2_. Another perspective suggests that once the cavitation bubbles burst, the extreme environment produces local temperatures high enough to decompose the sonosensitizers, breaking some chemical bonds. This process causes the pyrolysis of water molecules, resulting in the production of hydroxyl radicals, or reactions with other endogenous substrates to produce additional free radicals (Figure 2B) [38]. Currently, the mechanism of ROS generation in SDT remains unclear and has not been accurately defined due to the complexity of sonodynamic processes. Significant efforts are needed to elucidate the underlying mechanism.

## 3. Application of Metalloporphyrins in PDT

### 3.1. The Characteristic of Metalloporphyrins as Photosensitizers

**Figure 3:**
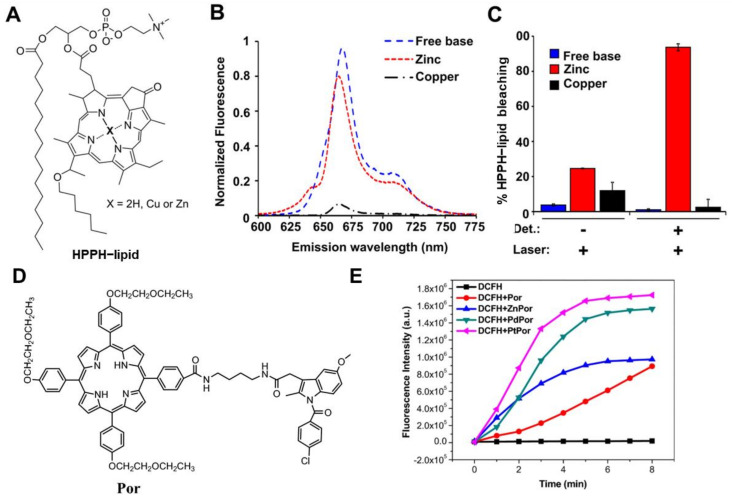
The photophysical properties and effect of different metal chelation on PoP^41^ and Por^42^ ligands. (**A**) Chemical structure of the HPPH lipids (PoP) examined. (**B**) Fluorescence emission spectra of an equal concentration of indicated PoP liposomes in phosphate-buffered saline. (**C**) Singlet oxygen generation was assessed indirectly by examining the increase in green fluorescence of a singlet oxygen sensor before and after laser irradiation. (**D**) Chemical structure of ZnPor, PdPor, and PtPor. (**E**) The fluorescence intensity detection at the characteristic peak of DCFH (525 nm) irradiated by different complexes as a function of irradiation time.

Since hematoporphyrin was used as the first photosensitizer, porphyrins and their derivatives have attracted great interest in pharmaceutical science [26,39]. The compounds are considered excellent photosensitizers due to their exceptional light stability and biocompatibility. When porphyrins coordinate with metal ions, the electronic structure of the central metal ion alters the spectroscopic properties of the entire molecule, leading to a broader absorption spectrum and enhanced light absorption capacity. For instance, metalloporphyrins can interact with the orbitals of surrounding molecules through the d-orbitals of the central metal, which facilitates more efficient electron transfer and promotes the occurrence of photo-induced reactions, which is important for dynamic therapy [40]. Meanwhile, metalloporphyrins often show good stability, biocompatibility, and tumor-targeting abilities. For example, Kevin et al. evaluated the effects of chelating copper or zinc into porphyrin liposomes (Cu-PoP and Zn-PoP) [41]. These complexes were chelated using copper (Cu)/zinc (Zn) ions with porphyrin–phospholipid (PoP), which was incorporated with 2-[1-hexyloxyethyl]-2-devinyl pyropheophorbide-a (HPPH)–lipid into a liposomal formulation (Figure 3A). Cu-PoP and Zn-PoP exhibit unique optical properties, such as high fluorescence and robust ROS generation capabilities (Figure 3B,C). Furthermore, compared to metal-free porphyrin liposomes, chelation with either Cu or Zn ions significantly enhanced their stability, biosafety, and biocompatibility. Additionally, these complexes increased effective drug delivery and release to tumor sites upon light irradiation, markedly improving tumor growth suppression. Wu et al. also prepared a series of metalloporphyrin–indomethacin conjugates (PtPor, PdPor, and ZnPor) tethered with poly(ethylene glycol) (PEG) chains (Figure 3D) [42]. Because of the heavy atom effect, the metal–porphyrin complexes exhibited a higher ^1^O_2_ quantum yield than free-base porphyrin (Por) (Figure 3E). The order of ^1^O_2_ yield of the synthesized metalloporphyrins was PtPor > PdPor > ZnPor > Por, and all these metalloporphyrin complexes showed better ^1^O_2_ generation than Por. MTT assays performed on HeLa cells revealed that these complexes exhibited minimal cytotoxicity in the dark, indicating their good biosafety. In contrast, under light exposure, PtPor showed the highest therapeutic efficacy. Schneider et al. synthesized several metalloporphyrin photosensitizers based on tetraplatinated (metallo)porphyrin [43]. After metal coordination, their water solubility and cellular penetration were significantly enhanced. Moreover, these photosensitizers exhibited excellent phototoxicity upon light irradiation, generating amounts of ROS that effectively induced tumor cell death while with good biosafety in the absence of light.

### 3.2. Assisted Enhanced PDT

**Figure 4:**
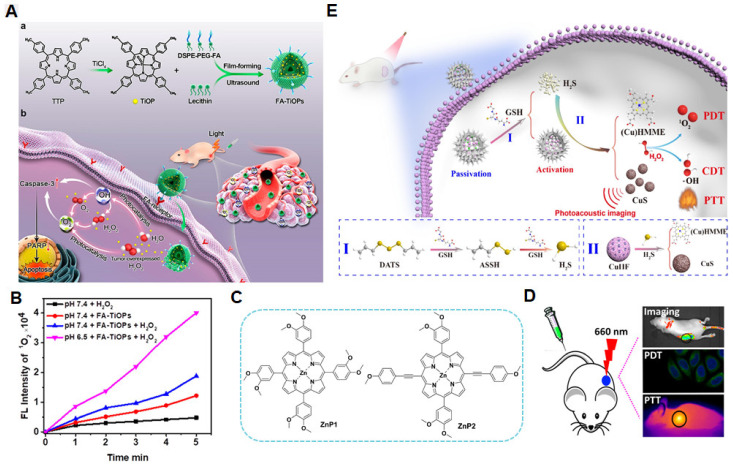
Different metalloporphyrins for enhanced antitumor PDT through assistant reaction and function. (**A**) TiOP-loaded liposome nanosystem (FA−TiOPs) to photocatalyze H_2_O and H_2_O_2_ for antitumor PDT (a) Preparation of FA–TiOPs. (b) Mechanism of in situ supplying ROS under FA–TiOPs photocatalysis [48]. (**B**) The enhanced ROS generation evaluation induced by FA-TiOPs under light irradiation. (**C**) The structure of ZnP1 and ZnP2 [49]. (**D**) Scheme of photothermal-assistant PDT-based ZnP2. (**E**) Scheme of self-supply CuS and (Cu)HMME for photothermal-assistant PDT. I: GSH triggered H_2_S generation; II: The H_2_S triggered CuS production and the resealse of metalloporphyrin ((Cu)HMMe) [50].

In addition to light-induced activation for ROS generation to kill tumor cells, metalloporphyrins often confer specific functions through the coordinated metal ions, which may provide unique antitumor properties that synergistically enhance therapeutic efficacy [44]. For instance, Mn(III)-coordinated ortho-N-alkyl- and N-alkoxyalkyl-porphyrins (MnPs) have been developed as superoxide dismutases to amplify oxidative stress in tumor cells. This elevated oxidative stress promoted protein oxidation and increased ROS levels, leading to apoptosis or necrosis of tumor cells, and thereby enhancing the effectiveness of cancer treatment [45]. Pt(IV) is highly toxic to cancer cells by blocking the replication and transcription of DNA, ultimately leading to cell death and synergistically enhancing the PDT effect [46]. In addition, metalloporphyrins can interact with small molecules or biomolecules within tumor cells, such as hydrogen peroxide, glutathione, and proteins. These interactions facilitate electron transfer, promoting the generation of ROS, thereby enhancing cytotoxicity and synergistically improving the overall antitumor efficacy [47]. Chen et al. designed a novel TiO–porphyrin nanosystem (FA-TiOPs) by encapsulating TiO–porphyrin (TiOP) in folate liposomes for antitumor PDT (Figure 4A) [48]. After the TiO group was inserted into the center of the porphyrin to obtain TiOP, folate-modified liposomes were used to load TiOP to improve the tumor-targeting delivery of metalloporphyrins. From the results, TiOP had excellent photocatalytic properties, and photocatalyzed H_2_O to split into ·OH radical, H_2_O_2_, and O_2_. Meanwhile, TiOP also catalyzed H_2_O_2_ and O_2_ to generate ^1^O_2_ under light excitation, further enhancing the anticancer effect (Figure 4B).

The light energy absorbed by metalloporphyrins can also be converted into heat, thereby synergistically enhancing tumor cell destruction. Ding et al. synthesized two Zn(II)-based metalloporphyrins (ZnP1 and ZnP2, Figure 4C) [49]. Both the complexes exhibited excellent photodynamic effects, with singlet oxygen quantum yields reaching 54% and 79%, respectively. Furthermore, ZnP2 nanoparticles demonstrated high photothermal conversion efficiency (~33.4%). As a result, ZnP2 showed a remarkable photothermal-enhanced PDT effect (Figure 4D). Beyond exogenous uptake of metalloporphyrins for photodynamic tumor therapy, metalloporphyrins can be synthesized in tumor sites. Tao et al. designed theranostic CuHF/DATS@HMON nanoplatforms consisting of hollow mesoporous organo-silica nanoparticles (HMON) loaded with CuHF and DATS (Figure 4E) [50]. Benefiting from the overexpressed glutathione (GSH) in the tumor environment, the −S-S-S- bond in DATS within the nanomaterials was cleaved, thus releasing H_2_S. The generated H_2_S subsequently reacted with CuHF to form CuS and (Cu)HMME. (Cu)HMME could be activated by light to produce ROS for tumor cell killing. CuS also absorbed light energy to convert heat for assistant enhancement of therapeutic outcomes.

### 3.3. Image-Guided PDT

**Figure 5:**
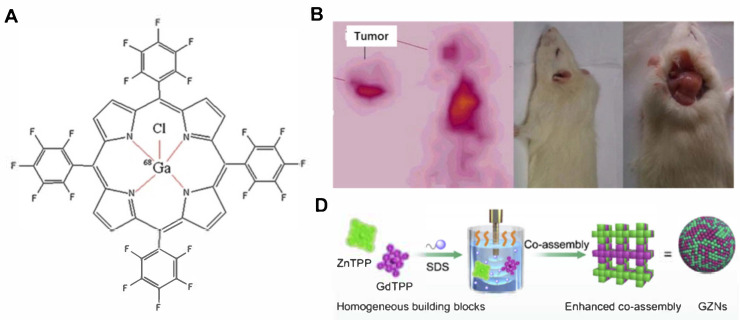

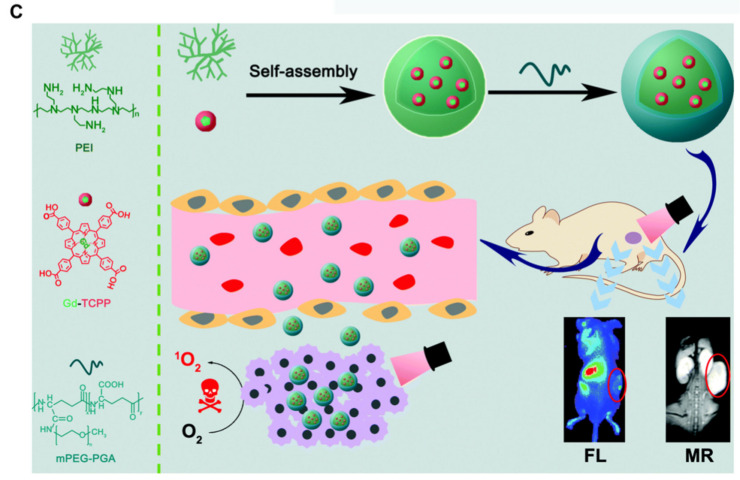
Imaging-guided precise tumor treatment with metalloporphyrin-based PDT. (**A**) The structure of ^68^Ga–porphyrin complex [53]. (**B**) MR imaging of ^68^Ga–porphyrin in tumor site. (**C**) Schematic illustration of the preparation of Gd–PNPs and their application in FL/MR imaging-guided PDT [55]. (**D**) Schematic illustrating the co-assembly of the nanocomposites by ZnTPP and GdTPP for MR/FL bimodal imaging-guided PDT [56].

Image-guided tumor therapy holds great promise for precise tumor treatment. By integrating imaging technologies with PDT, it allows for accurate localization of tumors and real-time monitoring of therapeutic effects. Metalloporphyrins, such as Mn- and Ga-porphyrins, have been used as magnetic resonance (MR) contrast agents [51,52]. Fazaeli et al. designed a ^68^Ga–porphyrin complex (Figure 5A) [53]. This radiolabeled porphyrin had strong PET imaging capability, integrating diagnosis and treatment using labeled metalloporphyrins (Figure 5B). Similarly, Hu et al. synthesized a water-soluble and tumor-targeted metalloporphyrin photosensitizer (Ga-4cisPtTPyP) containing Ga(III) and Pt(II) ions [54]. This photosensitizer showed a high singlet oxygen yield (Φ∆) and significant photocytotoxicity. Guided by MR imaging, Ga-4cisPtTPyP almost completely inhibited tumor growth over two weeks in an in vivo PDT assay. Treatment guided by multimodal imaging will increase the accuracy of therapy. Chen et al. prepared metalloporphyrin (Gd-TCPP)-based nanotheranostics (Gd-PNPs), realizing efficient PDT under dual-model guidance of fluorescence (FL) and MR imaging (Figure 5C) [55]. Notably, the multifunctional Gd–TCPP served triple functions in Gd–PNPs as an FL imaging probe, MR contrast agent, and PS for PDT. Cellular PDT experiments confirmed that Gd–PNPs exhibited good tumor cell ablation efficacy under light irradiation. The in vivo study proved that Gd–PNPs showed excellent PDT effect in CT26 tumor-bearing mice guided by FL/MR imaging. Except for single metalloporphyrins for image-guided PDT, metalloporphyrin complexes have also been developed for this purpose. Wang et al. prepared multifunctional metalloporphyrin nanocomposites (GZNs) by combining gadolinium porphyrin (GdTPP) as a contrast agent and zinc porphyrin (ZnTPP) as a photosensitizer (Figure 5D) [56]. The nanocomposites were further coated with tumor cell membranes to construct biomimetic nanomaterials (mGZNs). Through immune evasion via homologous cancer cell membrane camouflage, the mGZNs significantly improved tumor-targeting efficiency. Under MR/FL image guidance, the nanocomposites achieved an integrated and visualized cancer therapy.

### 3.4. Metalloporphyrin Frameworks for PDT

In addition to metalloporphyrin molecules, nanoscale porphyrin-based metal–organic frameworks (MOFs) have gained great attention as promising candidates for tumor therapy due to their features of large surface, high stability, apposite porosity, and good biocompatibility [57,58]. Zhao et al. synthesized a Ga−porphyrin MOF nanosheet through coordination between gadolinium(III) ions and 4,4,4,4-(porphine-5,10,15,20-tetrayl)tetrakis (benzoic acid) (TCPP) [59]. The photosensitive activity and relaxation rate of the Gd-TCPP were improved compared to both metal ions and organic ligands, realizing enhanced performance in magnetic resonance imaging and photodynamic therapy. Due to the high diffusion rates of oxygen and ROS within the porous material, MOFs allow for highly efficient PDT. Liu et al. assembled doxorubicin (DOX)-encapsulated zeolitic imidazolate frameworks (ZIF-8) on the surface of Zr(IV)-based porphyrinic MOFs [60]. The metalloporphyrinic MOFs harvested photons to produce plentiful ROS, thus leading to amplified chemo/PDT therapeutic efficacy. Similar phenomena were observed in other porphyrinic MOFs [61].

From the above research, it is clear that the insertion of different metal elements into the porphyrin center can not only alter its optical properties and enhance the antitumor effect but also reduce skin toxicity and improve biocompatibility [62]. Additionally, by selecting metal ions with specific functions (such as imaging or chemotherapy) to chelate porphyrin rings, the antitumor effect can be significantly improved through combined diagnosis or multimodal therapy [29]. Metalloporphyrin-based MOF showed potential application of PDT due to their porous property and biocompatibility and biodegradability [58]. Despite the potential benefits of metalloporphyrins in PDT, the limited penetration depth of visible light has restricted their widespread application, especially in deep-seated tumors, such as in liver cancer, kidney cancer, and glioma [63,64,65]. How to improve the PDT effect through structural modification of metalloporphyrins or optical technology is a big challenge.

## 4. Application of Metalloporphyrins in SDT

### 4.1. The Characteristics of Metalloporphyrins Chelated by Different Metal Centers for SDT

SDT is developed on the basis of PDT, aiming to address the inherent defect of light and improve deep penetration using ultrasound (US). Similar to PDT, SDT mainly generates ROS to kill tumor cells. Due to the deep penetration of US waves, it will create a broader application prospect for dynamic therapy [66,67,68]. Since hematoporphyrin, which is the first commercially available photosensitizer, was found to significantly kill various cancer cells after ultrasound irradiation, it paved a way for the application of porphyrins and their derivatives in SDT.

**Figure 6:**
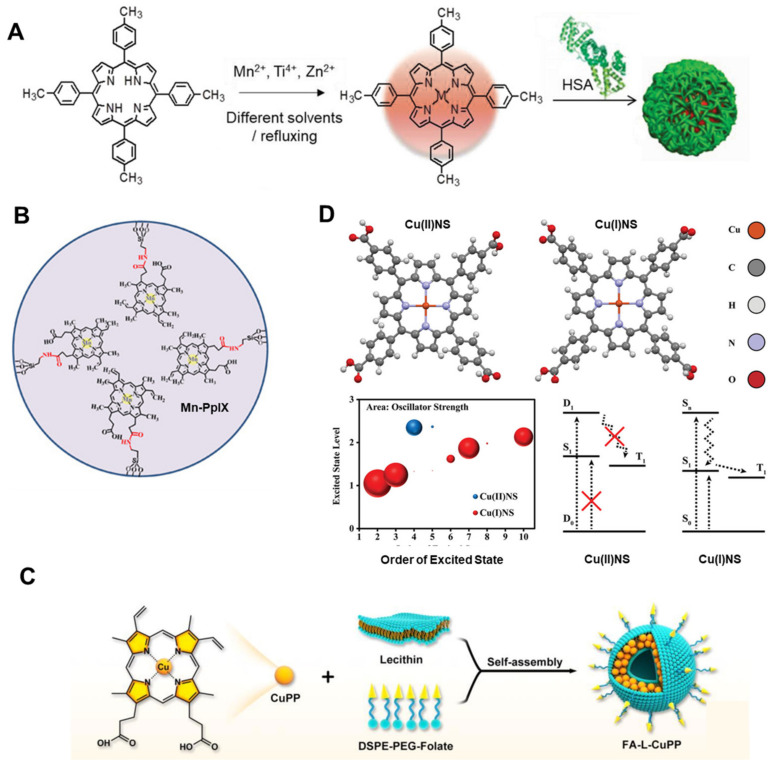
The SDT effects of some metalloporphyrins with different metal centers and porphyrin ligands. (**A**) Scheme illustrating the synthesis of MTTP complexes with different metal centers and the corresponding nanocomplexes of HSA for antitumor SDT [73]. (**B**) Structural illustration of Mn-PpIX-based sonosensitizer for antitumor SDT [74]. (**C**) Schematic illustration of the synthesis of FA–L–CuPP [75]. (**D**) The mechanism of Cu(II)NS and Cu(I)NS for SDT [76].

Due to the special advantages of metal ions (such as low phototoxicity, high stability, and functional diversity), a variety of metalloporphyrins have also been developed as sonosensitizers [22,69]. ATX-70, a Ga–porphyrin complex, was found to produce significant antitumor effects with both plane waves and focused waves while avoiding damage to surrounding tissues [70,71]. Giuntini et al. demonstrated that the ultrasound activity of metalloporphyrins largely depends on the presence of metal ions [72]. Therefore, different metal centers in metalloporphyrins exhibit a variety of properties under ultrasound irradiation. Ma et al. synthesized three metalloporphyrins (MnTTP, ZnTTP, and TiOTTP) with different metal coordination as sonosensitizers to investigate the influence of metal centers on SDT (Figure 6A) [73]. These metalloporphyrins were encapsulated with human serum albumin (HSA) to form nanoparticles (NPs), enhancing their biocompatibility and tumor-targeting ability. The results showed that these metalloporphyrins could all produce ROS under ultrasound irradiation. Moreover, they could be remotely activated to generate ROS over a distance of 10 cm, demonstrating the excellent deep penetration ability of ultrasound. Additionally, the study proved that different metal centers greatly influence the ultrasound response. Among the three, MnTTP had the best ROS generation effect under ultrasound, which can be explained by its high occupied molecular orbital (HOMO) and lowest unoccupied molecular orbital (LUMO) gap. Similarly, Huang et al. anchored a mesoporous hollow structure with Mn–protoporphyrin IX (Mn-PpIX) onto its surface as a sonosensitizer, and demonstrated highly effective antitumor SDT activity and good MR imaging, achieving image-monitored tumor growth inhibition (Figure 6B) [74].

In addition to Mn ion chelation to porphyrins, Cu–porphyrin (CuPP) has also been explored in antitumor SDT. Ma et al. used an urchin-shaped Cu–porphyrin (CuPP) liposome nanosystem (FA–L–CuPP) as a sonosensitizer (Figure 6C) [75]. FA–L–CuPP showed good tumor-targeting ability and ultrasound-mediated ROS generation. Molecular orbital distribution calculations indicated that strong intramolecular charge transfer of CuPP under US irradiation afforded energy to the surrounding O_2_ and H_2_O to generate ROS. Wang et al. further reported a Cu–porphyrin sonosensitizer (Cu(II)NS) consisting of Cu^2+^, TCPP, and poly(ethylene glycol) (PEG) (Figure 6D) [76]. They proved that overexpressed glutathione in the tumor could reduce Cu^2+^ to generate Cu(I)NS, leading to a singlet ground state, enhancing the antitumor SDT effect.

### 4.2. Synergistically Enhanced SDT Effect by Modulating Tumor Microenvironment

**Figure 7:**
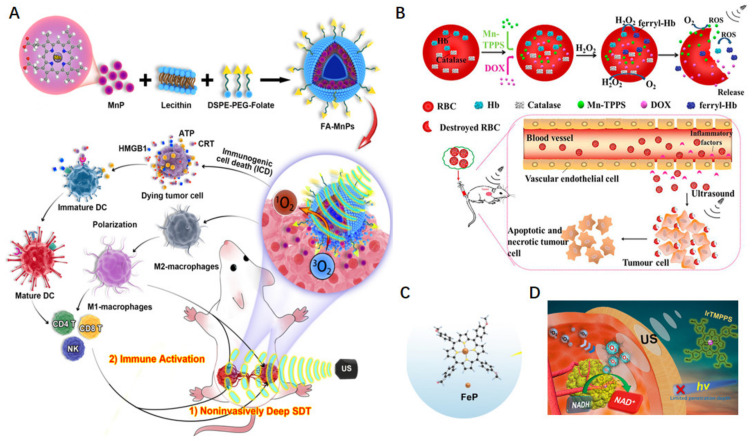
Tumor microenvironment adjustment relevant to SDT effect. (**A**) MnP-mediated SDT-ICD antitumor [78]. (**B**) Schematic of preparation and oxygen-enhanced SDT based on DOX/Mn-TPPS@RBCs [79]. (**C**) Oxygen-enhanced SDT based on Fe–porphyrin [80]. (**D**) Intracellular NADH oxidization for enhanced SDT effect [81].

Specific features of tumor microenvironments (such as immunosuppression and hypoxia) also influence antitumor effects. Adjusting the environment for more favorable conditions can improve therapy treatment [77]. Chen et al. discovered that ultrasound not only activates depth-enriched MnPs in tumors but also triggers a systemic immune response to synergistically prevent tumor recurrence [78]. They designed a multifunctional nanosonosensitizer (FA-MnPs) by encapsulating MnPs into folate–liposomes, a popular drug delivery carrier in cancer treatment, to increase the accumulation of drugs in tumors (Figure 7A). Under US treatment, FA-MnPs exhibited excellent US-mediated deep response and induced abundant ^1^O_2_ generation. Furthermore, FA-MnPs showed excellent suppression of superficial and deep-seated tumors in mice, providing effective in vivo SDT. MnP-mediated SDT also induced the re-polarization of macrophages from the immunosuppressive M2 type to the antitumor M1 type, and elicited immunogenic cell death (ICD) to synergistically suppress tumor growth and recurrence. To conquer hypoxia in tumors, Du et al. used an oxygen self-production red blood cell (RBC) carrier system by encapsulating a hydrophilic sonosensitizer (meso-tetra (4-sulfonatephenyl) porphyrinatemanganese(III) complex, MnTPPs), doxorubicin (DOX), and ferryl-hemoglobin (ferryl-Hb) inside RBCs (DOX/Mn-TPPs@RBCs, Figure 7B) [79]. Mn-TPPs not only enhanced the signal intensity of MR imaging at the tumor site but also acted as a sonosensitizers for SDT. Furthermore, by leveraging the oxygen-carrying capacity of ferryl-Hb, DOX/Mn-TPPs@RBCs overcame tumor hypoxia and improved the efficacy of SDT, realizing imaging-guiding triplex cancer therapy. Similarly, Yin et al. developed an O_2_-supplying nanosonosensitizer (FePO_2_@HC) by encapsulating Fe–porphyrin (FeP) and O_2_ in human serum albumin (Figure 7C) [80]. In FePO_2_@HC, dioxygen was adducted to FeP. Experimental results showed that FePO_2_@HC rapidly released O_2_ in a deoxygenated PBS solution, suggesting the selective release of O_2_ in hypoxic environments. With the cutting function of CLG on the collagen fibers in the tumor, the FePO_2_@HC effectively permeated into tumors and generated ROS to inhibit tumor growth. Xie et al. designed an iridium(III)–porphyrin sonosensitizer (IrTMPPS) for SDT (Figure 7D) [81]. IrTMPPS generated ample ^1^O_2_ under US irradiation. Moreover, IrTMPPS sonocatalytically oxidized intracellular NADH, which would enhance SDT efficiency by breaking the redox balance in the tumor.

### 4.3. Metalloporphyrin Frameworks as Sonosensitizers for Enhanced Antitumor Effect

MOFs can integrate the merits of small organic and inorganic sonosensitizers and avoid bleaching and degradation problems. Meanwhile, the porous structure substantially enlarges the specific surface area, which enhances the interaction between ultrasound waves and the MOF structures, promoting ROS production. Bao et al. prepared a metalloporphyrin-based PCN-224 MOF nanosonosensitizer integrated with platinum (Pt) NPs as well as glucose oxidase (GOx) [82]. The loaded GOx could consume glucose, achieving a tumor starvation therapy effect. The amplified synergistic therapy of SDT and starvation therapy efficiently inhibited tumor growth. Similarly, Jia et al. fabricated a core–shell heterostructure sonosensitizer based on a meso-tetra (4-carboxyphenyl) porphine (TCPP) porphyrin metal–organic framework (PCN-224(Fe)), keeping NaErF4:Yb@NaLuF4 nanoparticles as the core for precise optical imaging at NIR IIb (≈1500–1800 nm) [83]. Coordination of Fe^3+^ into the macrocycle of TCPP at the MOF shell highly increased the triplet state (T1) oxygen quenching, substantially promoting singlet oxygen generation and realizing dynamic monitoring of SDT processes. Compared to control groups, the in vitro and in vivo experiments both confirmed effective ROS generation and resulted in a sixfold volume reduction in brain gliomas. In other research, Niu et al. provided an effective strategy combining SDT and chemodynamic therapy (CDT) for leptomeningeal carcinomatosis (LMC) using a metalloporphyrin (iron(III) *meso*-tetra (4-carboxyphenyl)porphine chloride, TCPP(Fe))-integrated zirconium (Zr)-MOF-based nanosystem and further modified it with an arginine–glycine–aspartate (RGD) peptide (denoted as MOF@MP-RGD), overcoming brain-related barriers to improve delivery [84]. In this nanosystem, bifunctional TCPP(Fe) was employed not only as a sonosensitizer for SDT but also as a Fenton-type catalyst for CDT. The installation of TCPP(Fe) inside the framework of Zr-MOF nanoparticles aimed to ameliorate the inherent shortcomings of porphyrin, such as instability, poor water solubility, self-aggregation, and nonspecific targeting. The MOF@MPRGD nanosystem simultaneously induced the efficient generation of ^1^O_2_ under US irradiation and catalyzed the conversion of endogenous H_2_O_2_ into •OH by Fenton-like reactions, both of which caused adequate oxidative damage to tumor cells. The injected nanosystems were metabolized via the liver and then excreted from the body, mainly through feces, avoiding long-term body retention and potential in vivo toxicity. Ultimately, the combination of SDT and CDT obviously suppressed LMC growth and prolonged the survival time of LMC mice with favorable biosafety. Additionally, accurate monitoring of cancer treatment is greatly valuable for improvement of the therapeutic effect.

## 5. Combined PDT and SDT Based on Metalloporphyrins

The combination of PDT and SDT (PSDT) has been developed in the past decade. PSDT plays a synergistic role with better treatment effect, smaller sensitizer doses, and fewer side effects. Most porphyrin-based sensitizers are suitable for PDT and SDT. Liu et al. explored the combined effects of SDT and PDT using sinoporphyrin sodium (DVDMS) on breast cancer [85]. In in vitro and in vivo experiments, intracellular ROS were significantly increased after PSDT, and DVDMS-SPDT exhibited much more cytotoxicity compared with either SDT or PDT alone, suggesting better therapeutic effects after the combination of SDT and PDT. Geng et al. used a manganese ion coordinated PpIX (protoporphyrin IX) polymer (MnPPs) as a sensitizer nanoplatform for efficient antitumor PSDT [86]. MnPPs showed effective ROS generation after 5 min of light/ultrasound irradiation. At the same time, benefiting from the MR imaging function of Mn ions, PSDT resulted in excellent inhibition of tumor growth. Similarly, Xu et al. synthesized metalloporphyrin nanoparticles with a controlled packing pattern through noncovalent interactions using Mn (II) 5,10,15,20-tetraksi (4′-ethynylphenyl) porphyrin (MnTEPP) as a building block. Then, they constructed plasmonic heterostructure nanocomposites (SMAH) by depositing Au nanoparticles on the surface of the SM and further modified these using hyaluronic acid (HA) to target tumors and improve water solubility [87]. The nanocomposites significantly augmented the separation of electrons (e^−^) and holes (h^+^) and the catalytic ability of the nanoparticles under US and NIR light irradiation, leading to increased ROS production. Therefore, the SMAH nanoplatform efficiently killed cancer cells and suppressed tumor growth through PSDT with no obvious side effects at the injected dose.

Despite the increasing application of metalloporphyrin complexes in dynamic therapies, most studies have primarily focused solely on their therapeutic functions as sensitizers. The PSDT modality is still in its preliminary stages. Further investigation is required to explore the combined treatment approach.

## 6. Pharmacokinetics of Metalloporphyrins in Cancer Therapy

The pharmacokinetics of metalloporphyrins directly impact their therapeutic efficacy, and it is crucial to maintain sufficient tumor accumulation while minimizing systemic toxicity. The pharmacokinetics of metalloporphyrins generally involve the following key qualities. (1) The presence of metal ions enhances the structural stability of metalloporphyrins, making many of these compounds relatively stable in vivo. This stability helps to maintain effective concentrations of the sensitizers within tumor tissues through the enhanced permeability and retention (EPR) effect. (2) Although the exact metabolic pathways are not fully understood, existing studies suggest that some metalloporphyrins are metabolized in the liver. Their clearance rates in the body are largely determined by their hydrophilicity and the nature of the metal ion. Hydrophilic metalloporphyrins typically have shorter half-lives and are excreted more rapidly, while lipophilic metalloporphyrins may be retained in tissues for longer periods. Different metal ions have varying charges, radii, and chemical affinities. Therefore, the type of the chelated metal ions also influences the distribution and metabolic rate of metalloporphyrins.

## 7. Conclusions and Perspectives

Metalloporphyrins have unique characteristics, such as photocatalysis, electrocatalysis, and biomimetic catalysis. Moreover, they possess potential biological properties, such as biocompatibility, effective tumor retention, and various mimicking of biological functions, making them extremely useful for biomedical applications, especially for PDT and SDT. Despite the growing number of impressive reports on metalloporphyrins, their biomedical applications in clinical settings are still in the early stages and face many challenges. First, in PDT, the depth of light penetration is limited, particularly for short visible wavelengths, making it difficult to effectively treat deep-seated tumors. Recently, new in vivo light source technologies (such as optical fibers and chemiluminescence) have emerged. Exploring how to use these technologies to enhance PDT effects is an attractive direction. In SDT, there are still limitations in treating specific tumor sites, and new in vivo technologies are also required. Second, metalloporphyrins have excellent photophysical and photochemical properties, which provide advantages for their application in PDT and SDT. However, phototoxicity is a severe problem due to the continuous accumulation of these sensitizers in the skin. Reducing dark toxicity with ensuring dynamic effects is an important research direction in the long term. Third, the stability and biocompatibility of metalloporphyrins in vivo directly affect their therapeutic efficacy and safety. Some metalloporphyrins may degrade or react with other molecules in the body, affecting their function. Ensuring their stability and compatibility is crucial for effective treatment. Finally, thorough and in-depth studies are necessary to understand the correlation between the fundamental parameters of metalloporphyrins and their biosafety, including biodistribution, pharmacokinetics, long-term toxicity, and clearance in vivo, to enable potential clinical translation. Moreover, benefiting from the ROS generation of metalloporphyrins under light/ultrasound irradiation, the combination of PDT and SDT may synergistically increase the production of ROS in various ways, which would kill tumor cells via multiple mechanisms and improve therapy effects. Overall, metalloporphyrins have great potential in tumor treatment, and significant progress is expected in the biomedical field in the future.
